# Automated Diagnosis and Phenotyping of Tuberculosis Using Serum Metabolic Fingerprints

**DOI:** 10.1002/advs.202406233

**Published:** 2024-08-19

**Authors:** Yajing Liu, Ruimin Wang, Chao Zhang, Lin Huang, Jifan Chen, Yiqing Zeng, Hongjian Chen, Guowei Wang, Kun Qian, Pintong Huang

**Affiliations:** ^1^ Department of Ultrasound in Medicine The Second Affiliated Hospital of Zhejiang University School of Medicine Zhejiang University Hangzhou 310009 P. R. China; ^2^ State Key Laboratory for Oncogenes and Related Genes School of Biomedical Engineering Institute of Medical Robotics and Med‐X Research Institute Shanghai Jiao Tong University Shanghai 200030 P. R. China; ^3^ Post‐Doctoral Research Center Zhejiang SUKEAN Pharmaceutical Co., Ltd Hangzhou 311225 P. R. China; ^4^ Research Center for Life Science and Human Health Binjiang Institute of Zhejiang University Hangzhou 310053 P. R. China

**Keywords:** diagnosis and phenotyping, drug resistant tuberculosis, nanoparticle enhanced laser desorption/ionization mass spectrometry, serum metabolic fingerprints, tuberculosis

## Abstract

Tuberculosis (TB) stands as the second most fatal infectious disease after COVID‐19, the effective treatment of which depends on accurate diagnosis and phenotyping. Metabolomics provides valuable insights into the identification of differential metabolites for disease diagnosis and phenotyping. However, TB diagnosis and phenotyping remain great challenges due to the lack of a satisfactory metabolic approach. Here, a metabolomics‐based diagnostic method for rapid TB detection is reported. Serum metabolic fingerprints are examined via an automated nanoparticle‐enhanced laser desorption/ionization mass spectrometry platform outstanding by its rapid detection speed (measured in seconds), minimal sample consumption (in nanoliters), and cost‐effectiveness (approximately $3). A panel of 14 m z^−1^ features is identified as biomarkers for TB diagnosis and a panel of 4 m z^−1^ features for TB phenotyping. Based on the acquired biomarkers, TB metabolic models are constructed through advanced machine learning algorithms. The robust metabolic model yields a 97.8% (95% confidence interval (CI), 0.964‐0.986) area under the curve (AUC) in TB diagnosis and an 85.7% (95% CI, 0.806‐0.891) AUC in phenotyping. In this study, serum metabolic biomarker panels are revealed and develop an accurate metabolic tool with desirable diagnostic performance for TB diagnosis and phenotyping, which may expedite the effective implementation of the end‐TB strategy.

## Graphical abstract

This study presents a novel metabolomics approach for TB detection and phenotyping using an automated nanoparticle‐enhanced laser desorption/ionization mass spectrometry platform. It identifies 14 biomarkers for TB diagnosis and 4 for phenotyping, constructing robust metabolic models achieving 97.8% and 85.7% AUC, respectively. This tool promises to enhance TB management.

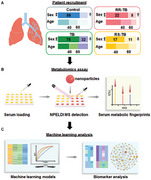

## 1 Introduction

Tuberculosis (TB) is a chronic communicable disease resulting from infection with *Mycobacterium tuberculosis (M.tb)*, which mainly affects the lungs.^[^ ^1^ ^]^ TB stands as the second most fatal infectious disease after COVID‐19 and kills 1.3 million people globally in 2022. With low cure rate, high mortality rate and strong infectivity, drug‐resistant TB imposes a heavy financial burden on patients and families.^[^ ^2^ ^]^ Precision point‐of‐care diagnostic and phenotypic tests are indispensable for widespread screening and treatment of TB, as well as for the effective implementation of the end‐TB strategy. However, the diagnosis and phenotyping of TB remain challenging due to the lack of a satisfactory integrated method.^[^ ^3^ ^]^

Presently, conventional TB diagnosis and phenotyping methods encompass etiological examination, immunological, and molecular biological examinations.^[^ ^4^ ^]^ Among these, sputum smear examination is expeditious but grapples with diminished sensitivity and specificity in the identification of TB.^[^ ^5^ ^]^ Mycobacterial culture, deemed the gold standard for TB diagnosis, is time‐consuming and fails to meet the demands of same‐day, large‐scale identification of TB patients in general hospitals.^[^ ^6^ ^]^ Traditional drug sensitivity test utilizes mycobacterium tuberculosis to assess its resistance to anti‐tuberculosis drugs.^[^ ^7^ ^]^ However, due to the lengthy culture cycle, rapid reporting of drug resistance in tuberculosis patients infected with *M.tb* is not feasible. Xpert MTB/rifampicin (RIF) resistance molecular testing enables automated diagnosis of both TB and RIF‐resistant TB (RR‐TB) simultaneously.^[^ ^8^ ^]^ Nevertheless, the application of this method is substantially hindered by its high cost ($ 9.98 per test), challenges related to specimen collection, and variable sensitivity in cases of extrapulmonary TB.^[^ ^9^ ^]^ To address these issues, there is an urgent need for advanced diagnostic tools that possess high specificity and sensitivity, rapid testing speed, and easy accessibility for TB diagnosis and phenotyping.^[^ ^10^ ^]^

Metabolomics, as a high‐throughput systems biology approach, provides valuable insights into the identification of differential metabolites for disease diagnosis and phenotyping.^[^ ^11^ ^]^ Serum metabolic fingerprints (SMFs), as the culmination of intricate biochemical reactions, mirror the metabolic pathways and processes occurring in both physiological and pathological states.^[^ ^12^ ^]^ In the case of TB, *M.tb* and drug‐resistant strains have the capability to invade various organs throughout the body and influence the host's metabolism.^[^ ^13^ ^]^ Specific SMFs have been associated with *M.tb* infection.^[^ ^14^ ^]^ By identifying and quantifying changes in metabolites of the patients, it offers valuable information for TB diagnosis and phenotyping.^[^ ^15^ ^]^ Presently, nuclear magnetic resonance (NMR) and mass spectrometry (MS) are the predominant methods used for metabolite measurement.^[^ ^16^ ^]^ NMR is suitable for the analysis of compounds containing hydrogen, while MS‐based metabolomics is more appropriate for the quantitative and qualitative analysis of numerous metabolites.^[^ ^17^ ^]^ With advances in nanotechnology, nanoparticle‐enhanced laser desorption/ionization (NPELDI) MS has become a powerful method for comprehensive label‐free metabolic profiling, owing to the strong affinity between nanoparticles and metabolites, high throughput, and high accuracy and stability, which brings new hope for simultaneous diagnosis and phenotyping of TB.^[^ ^18^ ^]^

To identify TB‐specific patterns, high‐dimensional and high‐throughput NPELDI MS data should be processed in an advanced quality, reproducibility, and speed manner.^[^ ^19^ ^]^ Machine learning has been successfully used to enhance data quality and applied to intelligent prediction and interpretation of high‐throughput MS data.^[^ ^20^ ^]^ For both TB diagnosis and phenotyping, advanced machine‐learning algorithms should be employed to construct reliable diagnostic models.^[^ ^21^ ^]^ Herein, we employed well‐designed ferric nanoparticles for NPELDI MS platform to automatically acquire MS data without the need for enrichment or purification of serum. By the combination of NPELDI MS with machine learning, we successfully extracted SMFs of trace samples and identified specific metabolic biomarker panels of TB patients. The robust diagnostic model achieved a 97.8% area under the curve (AUC) in TB diagnosis and a 85.7% AUC in TB phenotyping, with satisfactory sensitivity and specificity.

## 2 Results and Discussion

### 2.1 Optimization of Ferric Nanoparticles for NPELDI MS

**Figure 1:**
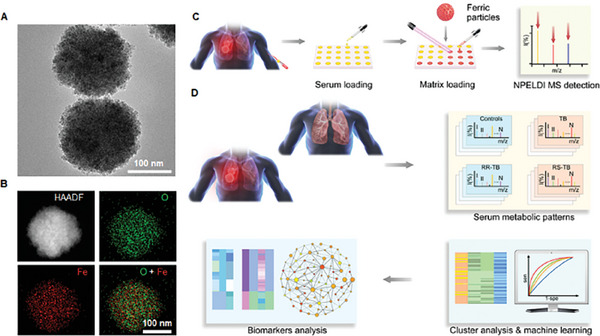
Schematics for the extraction of serum metabolic fingerprints (SMFs) for tuberculosis (TB) diagnosis and phenotyping by machine learning. A) TEM and B) High‐angle annular dark‐field (HAADF) image and elemental mapping of O (green) and Fe (red) for ferric nanoparticles. C) Experimental workflow of ferric nanoparticle enhanced laser desorption/ionization (NPELDI) mass spectrometry for SMFs extraction. D) Serum biomarkers for TB and RR‐TB screening and functional analysis determined by machine learning. The scale bar is 100 nm in A) and B).

We developed a NPELDI MS microarray utilizing ferric nanoparticles as a matrix, enabling reproducible, sensitive, and selective SMFs of samples. The ferric nanoparticles were synthesized utilizing a modified low‐cost solvo‐thermal technique.^[^ ^22^ ^]^ This method yielded approximately 0.8 g of ferric nanoparticles in a single synthesis, which can be utilized to test approximately 10^6^ samples for large‐scale clinical applications. TEM and SEM elucidated the polycrystalline structure and uneven surface of ferric nanoparticles, which agreed with the dynamic light scattering (DLS) results (≈250 nm diameter, polydispersity index (PDI) of 0.115) (**Figure** **1**A; Figure S1, Supporting Information). Figure S2 (Supporting Information) showed good water dispersibility of ferric nanoparticles.

The rough surface of ferric nanoparticles enhanced the adsorption of small molecular metabolites. The diffraction pattern was obtained from selected area electron diffraction (SAED), which displayed the crystalline structure of ferric nanoparticles (Figure S3, Supporting Information). The zeta potential of ferric nanoparticles was −22.1 mV, facilitating cation adduction through electrostatic attraction (Figure S4, Supporting Information).^[^ ^23^ ^]^ The UV–vis spectra in Figure S5 (Supporting Information) demonstrated a strong absorption in the range of 300–800 nm. The ferric nanoparticles, with specific ultraviolet absorption, could be easily excited by a 355 nm laser for NPELDI MS. Elemental mapping of Fe, O, and Fe+O validated the ferric nanoparticles (Figure 1B).

We then conducted comparisons of LDI MS results using 1,5‐diaminonaphthalene (1,5‐DAN), α‐cyano‐4‐hydroxycinnamic acid (CHCA), and 2,5‐dihydroxybenzoic acid (DHB), revealing either significant interference in the low mass range (100–400) or limited sensitivity in the analysis of standard metabolite mixtures (alanine (Ala), lysine (Lys), arginine (Arg), glucose (Glc), and sucrose (Suc)) (Table S1, Supporting Information). However, we acquired distinct MS from standard metabolite mixtures using ferric nanoparticles, demonstrating the superiority of our approach (Figure S6, Supporting Information). Hence, the ferric nanoparticles exhibited excellent water dispersibility as a matrix, a negatively charged surface facilitating cation adduction, high light absorption for efficient laser energy transfer, and optimal compositions suitable for NPELDI MS applications.

### 2.2 Metabolite Profiling in Serum Samples

**Figure 2:**
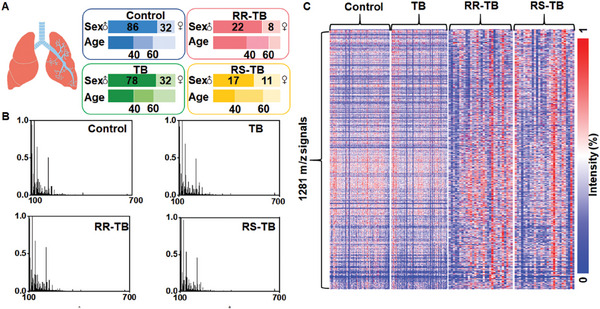
A) Demographics of the 228 patients from whom clinical samples were obtained for both the discovery and validation cohorts show that age and sex were matched with no significant differences (p > 0.05). B) Representative mass spectra were obtained from a healthy control, a TB patient, an RR‐TB patient, and an RS‐TB patient by NPELDI MS. C) SMFs were obtained from raw mass spectra of 118 HC, 110 TB patients, 30 RR‐TB patients, and 28 RS‐TB patients, respectively.

228 serum samples were collected, comprising 110 from TB patients and 118 from healthy controls (HC). There were 30 patients with RR‐TB and 28 patients with RIF‐sensitive TB (RS‐TB) in all patients with TB. Patients with TB were diagnosed by professional clinicians according to culture, smear, imaging examinations, and molecular diagnosis results.^[^ ^24^ ^]^ All serum samples used in the diagnosis model were randomly split into nonoverlapping discovery (n = 90:81, TB/HC) and independent validation (n = 20:37, TB/HC) cohorts. Serum samples in the RR‐TB model were split into nonoverlapping discovery (n = 23:20, RR‐TB/RS‐TB) and independent validation (n = 7:8, RR‐TB/RS‐TB) cohorts. No significant statistical differences were found in age or sex between TB patients and the HC, RR‐TB, and RS‐TB cohorts (p > 0.05; **Figure** **2**A; Tables S2 and S3, Supporting Information). After preprocessing the raw MS data (with ≈120000 data points before extraction), 1281 m z^−1^ signals with the highest localized intensity were extracted and identified as SMFs (Figure 2B,C).

### 2.3 Machine Learning for TB Diagnosis and Phenotyping

**Figure 3:**
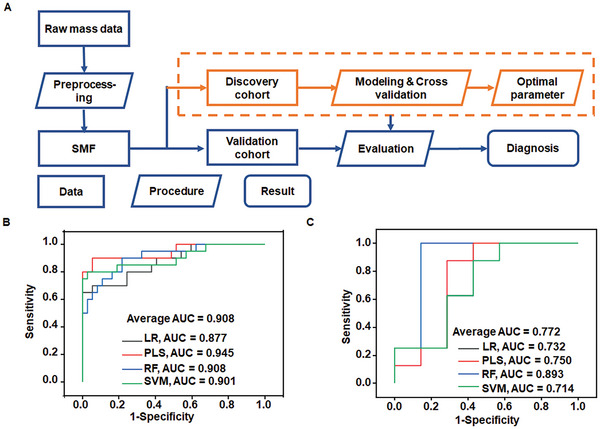
A) Workflow for TB diagnosis using serum metabolic fingerprinting and machine learning. Receiver operating characteristic (ROC) curves using logistic regression (LR, black), support vector machine (SVM, red), partial least squares (PLS, blue) regression, and random forest (RF, green) to distinguish between B) TB patients and healthy controls and C) RR‐TB and RS‐TB patients in the validation cohort.

To evaluate the diagnostic efficacy of SMFs, we employed machine learning within the same workflow for the diagnosis and phenotyping of TB (**Figure** **3**A). We applied regularization to ensure the reliability of receiver operating characteristic (ROC) curve results. The 95% confidence interval (CI) of the AUC was obtained through bootstrap. The independent test was applied to show the consistent diagnostic performance of TB metabolic models. The AUC via different algorithms of logistic regression (LR)/partial least squares (PLS)/random forest (RF)/support vector machine (SVM) was calculated as 0.877 (sensitivity 65% and specificity 100%)/0.945 (sensitivity 90% and specificity 94.6%)/0.908 (sensitivity 90% and specificity 78.3%)/0.901 (sensitivity 80% and specificity 97.3%) in the validation cohort, respectively (Figure 3B; **Table** **1**). Our machine learning‐aided diagnostic approach exhibited a remarkable performance for TB diagnosis with a mean AUC of approximately 0.908, a mean sensitivity of about 81.3%, and a mean specificity of roughly 92.6%. For TB phenotyping, we employed machine learning to extract SMFs of RR‐TB and RS‐TB patients from raw MS data. Intriguingly, the RF diagnostic model exhibited the highest AUC of 0.893 (95% CI, 0.860‐0.921) in the validation cohort, featuring an impressive sensitivity of 100% and a specificity of 85.7% (Figure 3C; **Table** **2**). Significantly, the cost for each test in the diagnosis of RR‐TB through our approach was merely $3, rendering it a more cost‐effective option for clinical RR‐TB screening.

**Table 1:** Comparison of the diagnostic performance of TB assessment based on different algorithms.

	Machine Learning	Cohorts	AUC[95% CI]	Sensitivity	Specificity
SMFs	LR	Validation	0.877 (0.839‐0.906)	0.650	1.000
		Discovery	1.000 (1.000‐1.000)	1.000	1.000
	PLS	Validation	0.945 (0.919‐0.967)	0.900	0.946
		Discovery	1.000 (1.000‐1.000)	1.000	1.000
	RF	Validation	0.908 (0.878‐0.932)	0.900	0.783
		Discovery	1.000 (1.000‐1.000)	1.000	1.000
	SVM	Validation	0.901 (0.863‐0.932)	0.800	0.973
		Discovery	1.000 (1.000‐1.000)	1.000	1.000

**Table 2:** Comparison of the diagnostic performance of RR‐TB assessment based on different algorithms.

	Machine Learning	Cohorts	AUC(95% CI)	Sensitivity	Specificity
SMFs	LR	Validation	0.732 (0.693‐0.775)	1.000	0.571
		Discovery	1.000 (1.000‐1.000)	1.000	1.000
	PLS	Validation	0.750 (0.707‐0.797)	0.875	0.714
		Discovery	1.000 (1.000‐1.000)	1.000	1.000
	RF	Validation	0.893 (0.860‐0.921)	1.000	0.857
		Discovery	1.000 (1.000‐1.000)	1.000	1.000
	SVM	Validation	0.714 (0.674‐0.751)	0.875	0.571
		Discovery	1.000 (1.000‐1.000)	1.000	1.000

### 2.4 Differential Metabolite Selection

**Figure 4:**
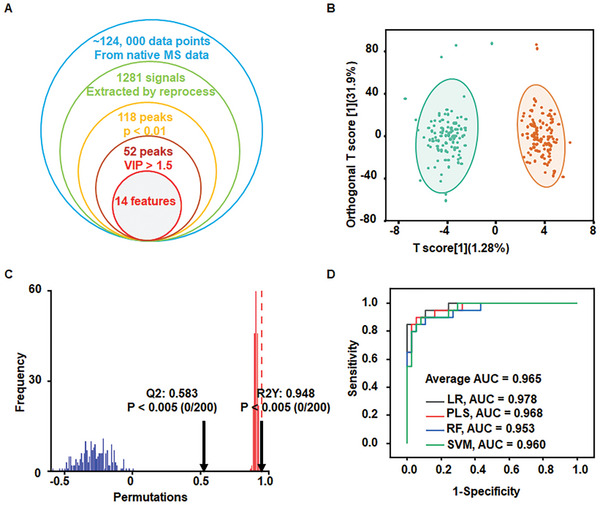
A) Venn diagram of 14 m z^−1^ differential features from 1281 metabolite signals in the serum of TB patients and healthy controls with *p* < 0.01 and VIP > 1.5. B) The OPLS‐DA model was able to separate the TB group from the healthy control group. C) The permutation test showed the prediction ability of the model with R^2^X (cum) = 0.529, R^2^Y(cum) = 0.948, and Q^2^(cum) = 0.583. D) Receiver operating characteristic (ROC) curves using logistic regression (LR, black), support vector machine (SVM, red), partial least squares (PLS, blue) regression, and random forest (RF, green) to distinguish between TB patients and healthy controls in validation cohorts based on selected metabolites.

**Figure 5:**
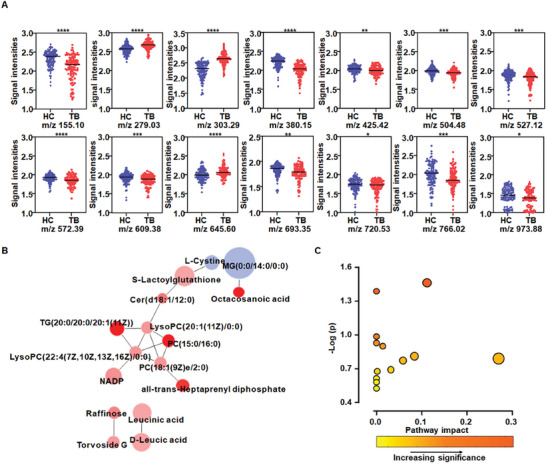
A) Scatter plots for log(10) standardized signal intensities of 14 metabolite features in healthy controls (blue) and TB patients (red). ^*^ denoted *p* < 0.05, ^**^ denoted *p* < 0.01, and ^***^ denoted *p* < 0.001 in Student's *t*‐test. B) Biomarkers differentially regulated between TB patients and the HC group. C) Pathway enrichment analysis of biomarkers from TB patients and HC group.

Differential metabolites were then screened as metabolic biomarkers for accurate TB diagnosis (**Figure** **4**A). Orthogonal partial least squares discriminant analysis (OPLS‐DA) was performed on 1281 metabolite signals. The OPLS‐DA revealed distinct discrimination between TB and HC groups (Figure 4B). A 100‐iteration permutation test validated the reliability of the OPLS‐DA model with R^2^X = 0.529, R^2^Y = 0.948, and Q^2^ = 0.583 (Figure 4C). The variable importance in the projection (VIP) is applied in reflecting the weight value of OPLS‐DA model variables.^[^ ^25^ ^]^ After screening with *p* < 0.01 and VIP > 1.5, differential metabolite signals were selected. Next, we compared the selected differential data with detailed information on metabolites from the Human Metabolome Database to identify metabolic biomarkers, of which 3 exhibited increased levels and 11 exhibited decreased levels (Table S4, Supporting Information; **Figure** **5**A). Based on the analysis of the biomarker panel, the machine learning‐assisted TB diagnosis model showed better diagnostic performance (the average AUC of approximately 0.965) than based on the analysis of all 1281 m z^−1^ features (the average AUC of 0.908) (Figure 4D; **Table** **3**). Interestingly, the LR classification model showed an impressive AUC of 0.978 (95% CI, 0.964‐0.986) in the validation cohort. The combinations of biomarkers significantly improved the accuracy of machine learning models.

**Table 3:** Comparison of the diagnostic performance of TB assessment using different data inputs and algorithms based on biomarkers.

	Machine Learning	Cohorts	AUC [95% CI]	Sensitivity	Specificity
Biomarker	LR	Validation	0.978 (0.964‐0.986)	0.850	1.000
		Discovery	0.970 (0.948‐0.984)	0.967	0.938
	PLS	Validation	0.968 (0.952‐0.980)	0.900	0.946
		Discovery	0.986 (0.978‐0.993)	0.967	0.963
	RF	Validation	0.953 (0.926‐0.968)	0.850	0.946
		Discovery	1.000 (1.000‐1.000)	1.000	1.000
	SVM	Validation	0.960 (0.938‐0.976)	0.900	0.912
		Discovery	0.978 (0.963‐0.990)	0.956	0.963

These biomarkers consisted of lipids (monoglyceride (MG), ceramide (Cer), phosphatidylcholine (PC), lysophosphatidylcholine (LysoPC), cholesteryl ester (CE) triglyceride (TG), and octacosanoic acid), amino acids (cystine, leucic acid), phosphate (all‐trans‐heptaprenyl diphosphate, nicotinamide adenine dinucleotide phosphate (NADP)), and basic compound (S‐lactoylglutathione). Metabolism is the biochemical foundation of all physiological processes.^[^ ^26^ ^]^ *M.tb* parasitizes host phagosomes and exploits the nutrients of the host to maintain its own reproduction and virulence.^[^ ^27^ ^]^ Host‐derived carbon sources and nitrogen sources, such as carbohydrates, lipids, and amino acids, are nutrients for *M.tb*.^[^ ^28^ ^]^ Among these biomarkers, MG, Cer, PC, LysoPC, CE, TG, and octacosanoic acid are lipids. Lipids account for the majority carbon sources, which can provide energy for the physiological activities of *M.tb*.^[^ ^29^ ^]^ Abnormal lipid changes in the serum of TB patients indicated that TB infection could result in the dysregulation of host lipid metabolism. Amino acids (cystine, leucic acid) that function as primary nitrogen sources have been proven to support *M.tb* growth in vitro.^[^ ^30^ ^]^ In addition, abnormal expression of phosphate (all‐trans‐heptaprenyl diphosphate, NADP) and a basic compound (S‐lactoylglutathione) were observed in TB patients. S‐lactoylglutathione can be converted into glutathione. Reduced glutathione concentration is associated with *M.tb*‐induced macrophage necrosis.^[^ ^31^ ^]^ The levels of NADP, as a key participant in the glutathione metabolism pathway, were reduced in the serum of TB‐infected patients.^[^ ^32^ ^]^

The AUC value achieved using analyses of key biomarkers was obtained and suggested that this approach could be used to distinguish between TB and HC with great classification abilities (Table S4, Supporting Information). Among the biomarkers, the highest AUC value of 0.907 (95% CI, 0.878‐0.933) was achieved in the analysis of MG (0:0/14:0/0:0), a glyceride, which exhibited significantly increased levels. Furthermore, an AUC value of 0.877 (95% CI, 0.854‐0.900) was observed in the analysis of S‐lactoylglutathione, a basic oligopeptide, which exhibited significantly decreased levels (Figure 5A). The biomarker‐biomarker interaction network was used to visualize the interactions between a panel of biomarkers for TB diagnosis (Figure 5B). Each node represented a biomarker. Each edge (node‐to‐node connections) represented the interactions between these biomarkers. Node colors indicated metabolite expression changes in the serum of TB patients relative to HCs, red indicated decreased levels and blue indicated increased levels. Metabolic pathways (Figure 5C) were analyzed using MetaboAnalyst 5.0.^[^ ^33^ ^]^ Seven metabolic pathways were obtained, as follows: 1) sphingolipid metabolism, 2) glycerophospholipid metabolism, 3) ether lipid metabolism, 4) pyruvate metabolism, 5) galactose metabolism, 6) glycerolipid metabolism, and 7) glutathione metabolism (Table S5, Supporting Information). The abnormal expression of Cer(d18:1/12:0) suggested differential regulation of sphingolipid metabolism with the most significant impact (0.270), and this process is essential for TB host cell infection.^[^ ^16^ ^]^ We also found that phosphatidylcholine (PC) and lysophosphatidylcholine (LPC) levels decreased significantly in the serum of TB patients, which reflected the differential regulation of glycerophospholipid metabolism in TB.^[^ ^34^ ^]^

For TB phenotyping, we also performed the same biomarker screening approach to reveal the metabolic differences between the RR‐TB and RS‐TB groups (Figure S7, Supporting Information). There were significant differences in 4 m/z features between the two groups. A new metabolic panel was generated for TB phenotyping. These biomarkers consisted of taurine, homocysteine, uric acid, and ascorbic acid (Table S6, Supporting Information). Taurine is a sulfur amino acid as homocysteine. It is a membrane stabilizer and antioxidant that has protective effects against hepatotoxicity induced by chemicals, such as the antituberculosis drugs isoniazid and rifampicin.^[^ ^35^ ^]^ Antitubercular therapy was reported to be related to homocysteinemia, which refers to the elevation of plasma/serum homocysteine levels.^[^ ^36^ ^]^ The high expression of taurine and homocysteine in the blood of RR‐TB patients may be associated with drug resistance. Enhanced diagnostic performance (average AUC of approximately 0.830) of TB phenotyping model was achieved based on 4 m/z differential features, in contrast to those relying on 1281 m/z signals (with an average AUC of approximately 0.772) (Figure S8; Table S7, Supporting Information). Four different metabolic pathways were identified, including (1) taurine and hypotaurine metabolism, (2) cysteine and methionine metabolism, (3) primary bile acid biosynthesis, and (4) purine metabolisms. Among these pathways, taurine and hypotaurine metabolism had the most important impact at 0.429 (Figure S9; Table S8). The impact of cysteine and methionine metabolism was 0.138.

## 3 Conclusion

This study has 4 limitations, including 1) Sample size needs to be expanded across diverse clinical settings and geographic regions, encompassing various ethnic populations to ensure real‐world applicability; 2) Integrating metabolic fingerprints with multimodal clinical indexes will enhance TB screening efficacy; 3) Further studies are necessary on multidrug‐resistant TB and extensively drug‐resistant TB to optimize the clinical application of our approach; and 4) Integrating automated diagnostic tools into routine clinical practice poses challenges in terms of workflow integration and physician acceptance.

In this study, we extracted SMFs using an automated NPELDI MS approach for TB, with high diagnosis speed (≈seconds), minimal sample consumption (≈nL), low‐cost test (≈ $3) and wide coverage of metabolites (molecular weight < 1500 Da). Serum metabolic biomarker panels were identified for TB diagnosis and phenotyping. Robust machine‐learning models were constructed with an AUC of 97.8% for TB diagnosis and an AUC of 85.7% for TB phenotyping. These findings offer a significant step forward in the automated, rapid, and precise identification of TB patients, particularly in areas with a high TB incidence. This approach not only enhances early detection but also contributes to the global effort to control and eventually eliminate TB.

## 4 Experimental Section

### Clinical Subjects

In total, 228 subjects were recruited from 2020 to 2021 in Hangzhou Red Cross Hospital and the Second Affiliated Hospital of Zhejiang University, including 110 patients with pulmonary TB and 118 healthy subjects. For pulmonary TB patients, the following diagnostic criteria should be fulfilled: A) smear‐positive sputum; B) cultures positive for *M.tb*; C) nucleic acid detection of *M.tb*; D) chest radiograph (X‐ray or CT scan); E) pulmonary histopathology diagnosis of TB. Patients with RR‐TB or RIF‐sensitive TB (RS‐TB) were diagnosed according to the following criteria: Patients diagnosed with TB were demonstrated in vitro by drug sensitivity testing or Xpert MTB/RIF to be resistant or sensitive to rifampicin. Subjects diagnosed with extrapulmonary TB, HIV, end‐stage renal disease, malignant neoplasms, respiratory disease, or other lung diseases were excluded from the study. The study was carried out under the declaration of Helsinki (1975) and approved by the Ethics Committee of Zhejiang University School of Medicine, China. Written informed consent was obtained from each participant in this study. This study was registered at ClinicalTrials.gov (NCT04490746).

### Clinical Serum Collection

From each subject, 2 mL of blood was drawn in a venous blood collection tube (BD Vacutainer SST II Advance, USA) with a Medical Record Number. After 30 minutes at room temperature (20–25 °C), the sample was centrifuged at 3500 rpm and 4 °C for 10 min. Transfer the serum to a plastic transport tube and immediately store the tube at −80 °C for the subsequent MS analysis. All operations were completed within 1 h.

### NPELDI MS Detection

In a well‐designed NPELDI MS study, ferric nanoparticles were used as a matrix (1 mg mL^−1^). The preparation of ferric nanoparticles was according to the Nature Protocol Exchange.^[^ ^37^ ^]^ Serum samples (1 µL) were diluted and spotted onto a polished plate, followed by drying at room temperature. Then, the matrix slurry (1 µL) was applied and air‐dried for NPELDI MS analysis. The AutoFlex mass spectrometer (Bruker, Germany) equipped with a Nd: YAG laser (wavelength = 355 nm, peak power <170 kw, pulse width >3 ns, pulse energy <500 µJ, average power <1.5 w) was used for recording mass spectra of biomolecules. The laser beam width is as low as 10 µm. Mass calibration was conducted using standard small metabolites for accurate mass measurement and the positive ion mode was applied in all MS experiments. The pulse frequency and number of laser shots for each analysis were set to 1 kHz and 2000, respectively. The acceleration voltage was set as 20 kV and the delay time was 200 ns.

### MS Data Processing and Machine Learning

MS data preprocessing was performed, including peak extraction, alignment, normalization, and standardization by a MATLAB “home‐built” code (R2016a, The Mathworks, USA). LR, RF, SVM, and PLS algorithms were implemented based on Python 3.6. ROC curves were generated using classification probabilities of TB patients versus healthy controls. The performance of classification models was evaluated by AUC, sensitivity, and specificity.

### Statistical Analysis

Multiple feature selection approaches were performed to avoid bias, including univariate analysis and multivariate statistical analysis methods. Univariate analyses were conducted using SPSS software (version 19.0, Chicago), including the Student's *t*‐test for comparing age and the Chi‐square test for comparing sex. The deLong test was used to compare different ROC curves. A p‐value below 0.05 was considered statistically significant. Figures were created using Origin 2021 (OriginLab) and GraphPad Prism 9 (GraphPad) software. OPLS‐DA was conducted using SIMCA 14.1 (Umetrics, Sweden). Metabolic pathways were analyzed by MetaboAnalyst 5.0 (http://www.metaboanalyst.ca). Cytoscape (https://cytoscape.org/) was used for visualizing complex metabolite interaction networks.

## Conflict of Interest

The authors declare no conflict of interest.

## Supporting information

Supporting Information

## Data Availability

The data that support the findings of this study are available from the corresponding author upon reasonable request.
