# Supplementary material for: Automated Diagnosis and Phenotyping of Tuberculosis Using Serum Metabolic Fingerprints
Source: Adv Sci (Weinh). 2024 Aug 19;11(39):2406233. doi: 10.1002/advs.202406233 (PMC11497029; doi:10.1002/advs.202406233)
Supplement: Supplementary file 1 — Supporting Information [file ADVS-11-2406233-s001.docx]

Supporting Information

**Automated Diagnosis and Phenotyping of Tuberculosis Using Serum Metabolic Fingerprints**

*Yajing Liu, Ruimin Wang, Chao Zhang, Lin Huang, Jifan Chen, Yiqing Zeng, Hongjian Chen, Guowei Wang*, Kun Qian*, Pintong Huang**

Dr. Y. Liu, Dr. C. Zhang, Dr. J. Chen, Y. Zeng, Dr. G. Wang, Prof. P. Huang

Department of Ultrasound in Medicine, The Second Affiliated Hospital of Zhejiang University School of Medicine, Zhejiang University, Hangzhou 310009, P. R. China.

E-mail: wangguowei@zju.edu.cn, huangpintong@zju.edu.cn

Prof. P. Huang

Research Center for Life Science and Human Health, Binjiang Institute of Zhejiang University, Hangzhou 310053, P. R. China

Dr. R. Wang, Dr. L. Huang, Prof. K. Qian

State Key Laboratory for Oncogenes and Related Genes School of Biomedical Engineering Institute of Medical Robotics and Med-X Research Institute, Shanghai Jiao Tong University, Shanghai 200030, P. R. China

E-mail: k.qian@sjtu.edu.cn

Dr. H. Chen

Post-Doctoral Research Center, Zhejiang SUKEAN Pharmaceutical Co., Ltd, Hangzhou 311225, P. R. China.

This section includes the following:

1. Figure S1-9

2. Table S1-8


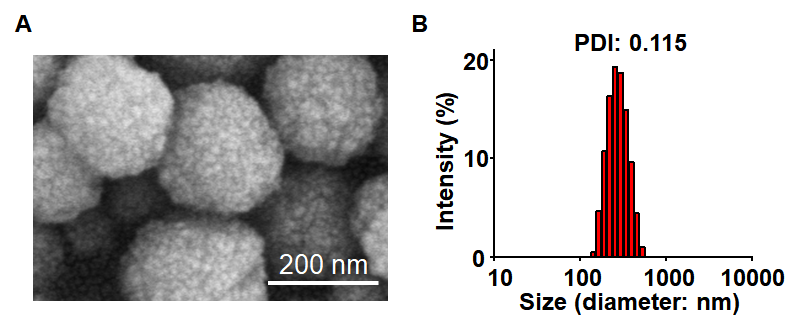


**Figure S1.** A) SEM image of ferric nanoparticles. B) Size distribution of ferric nanoparticles by dynamic light scattering (DLS). The scale bar is 200 nm in (A).


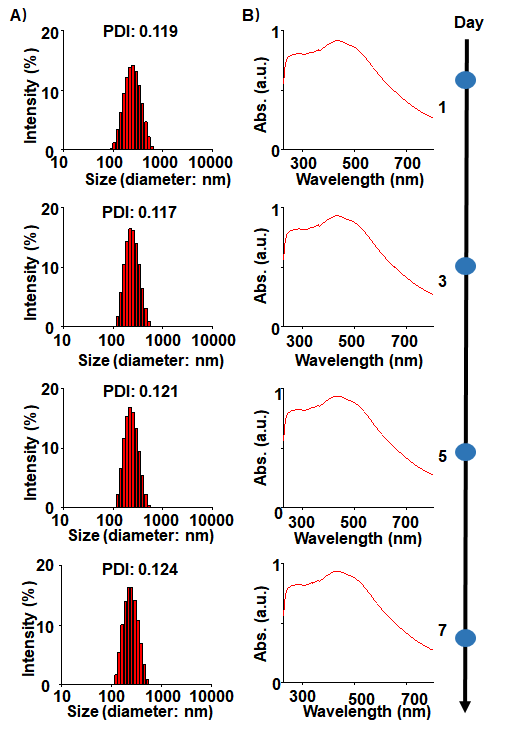


**Figure S2.** Water dispersibility of ferric nanoparticles. A) Size distribution and B) UV-vis spectrum of ferric nanoparticles in aqueous solution during one week at the time point of 1, 3, 5, and 7 day.


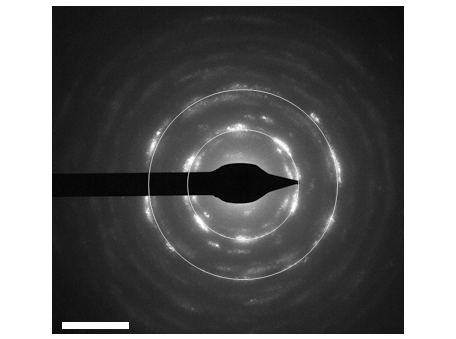


**Figure S3.** Selected area diffraction (SAED) image of the ferric nanoparticles. The scale bar is 5 nm^-1^.


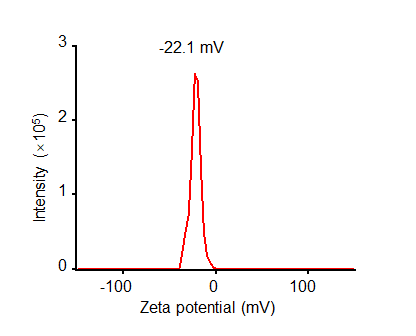


**Figure S4.** Zeta potential of ferric nanoparticles.


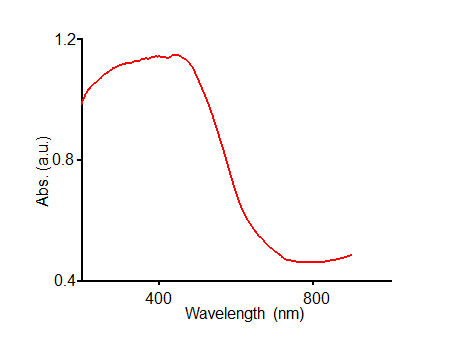


**Figure S5.** Ultraviolet-visible spectra of ferric nanoparticles.


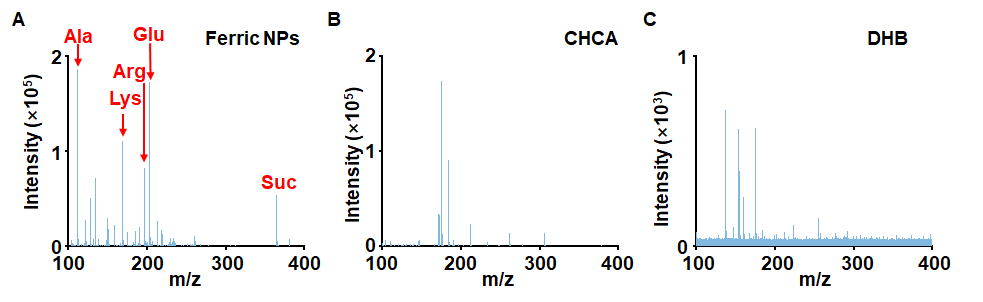


**Figure S6.** Laser desorption/ionization (LDI) mass spectra of standard metabolite mixture (1 mg mL^−1^ of alanine (Ala), lysine (Lys), arginine (Arg), glucose (Glc), and sucrose (Suc)) using A) ferric nanoparticles, B) α-cyano-4-hydroxycinnamic acid (CHCA), and C) 2,5-dihydroxybenzoic acid (DHB).


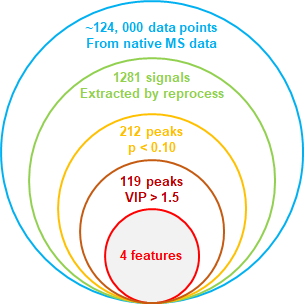


**Figure S7.** Venn diagram of 4 m/z differential features from 1281 metabolite signals in serum of RR-TB and RS-TB patients with variable importance in projection (VIP) > 1.5 and p < 0.10.


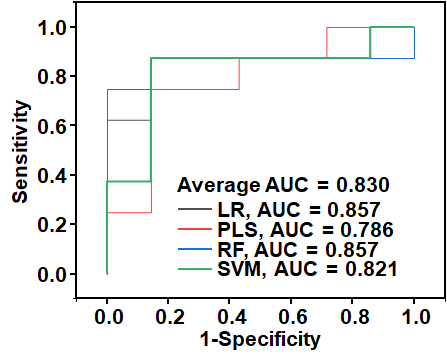


**Figure S8.** ROC curves using LR (black), SVM (red), PLS (blue), and RF (green) to distinguish RR-TB from RS-TB in validation cohort (n = 58; 30/28, RR-TB/RS-TB) relying on selected metabolites.


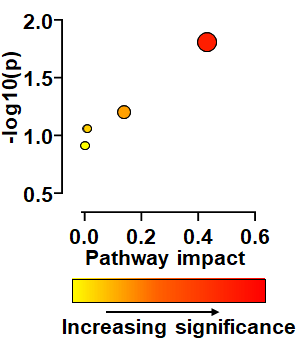


**Figure S9.** Potential pathways differentially regulated in RR-TB patients, compared to RS-TB patients.

**Table S1.** Limit of detection of metabolites detected by different matrices.

| **Analytes** | **Detection limit (pmol)** | | | |
| --- | --- | --- | --- | --- |
|  | **Ferric nanoparticles** | **CHCA** | **DHB** | **1,5-DAN** |
| Valine | 85.4 | 8540 | 854 | **-** |
| Lysine | 684 | - | - | **-** |
| Alanine | 11.22 | 1122 | 112.2 | **-** |
| Glucose | 5.55 | 55.5 | 555 | 55.5 |
| Sucrose | 29.2 | - | - | **-** |

**Table S2.** Age and sex characteristics of healthy controls and TB patients.

| **Characteristics** | | **Discovery** | | | | |  | | **Validation** | | | | | |  |
| --- | --- | --- | --- | --- | --- | --- | --- | --- | --- | --- | --- | --- | --- | --- | --- |
|  |  | **Controls**  **(n=81)** | | **TB**  **(n=90)** | | **P value** | |  | | **Controls**  **(n=37)** | | **TB**  **(n=20)** | | **P value** | |
| Age  (Mean ± SD) | | 48.46 ± 18.90 | 49.72 ± 18.96 | | | 0.663^a)^ | |  | | 53.54 ± 19.32 | 51.20 ± 19.07 | | | 0.663^a)^ | |
| Sex | |  |  | |  | | |  | |  |  | |  | | |
|  | Male | 60 | 61 | | 0.366^b)^ | | |  | | 26 | 17 | | 0.363^b)^ | | |
|  | Female | 21 | 29 | |  | | |  | | 11 | 3 | |  | | |

a) p value was calculated using a two-sided Student's t-test; b) p value was determined via a Chi-square test.

**Table S3.** Age and sex characteristics of RR-TB patients and RS-TB patients.

| **Characteristics** | | **Discovery** | | | |  | | **Validation** | | | |  |
| --- | --- | --- | --- | --- | --- | --- | --- | --- | --- | --- | --- | --- |
|  |  | **RR-TB**  **(n=23)** | **RS-TB**  **(n=20)** | **P value** |  | | **RR-TB**  **(n=7)** | | **RS-TB**  **(n=8)** | **P value** | | |
| Age  (Mean ± SD) | | 43.35 ± 14.05 | 47.90 ± 14.52 | 0.303^a)^ |  | | 48.43 ± 16.22 | | 52.75 ± 16.43 | | 0.618 ^a)^ | |
| Sex | |  |  |  |  | |  | |  | |  | |
|  | Male | 18 | 11 | 0.104 ^b)^ |  | | 4 | | 6 | | 0.608 ^b)^ | |
|  | Female | 5 | 9 |  |  | | 3 | | 2 | |  | |

a) p value was calculated using a two-sided Student's t-test; b) p value was determined via a Chi-square test.

**Table S4.** 14 m/z signals selected as key metabolite features for differentiating TB patients from healthy controls.

| **ID** | **m/z** | **P value** | **AUC** | **Potential biomarkers** | **Regulated expression** | |
| --- | --- | --- | --- | --- | --- | --- |
| 1 | 155.102 | 5.25E-09 | 0.762  (0.732-0.801) | D-Leucic acid/Leucinic acid | | ↓ |
| 2 | 279.033 | 4.09E-09 | 0.780  (0.752-0.826) | L-Cystine | | ↑ |
| 3 | 303.288 | 2.84E-25 | 0.907  (0.878-0.933) | MG(0:0/14:0/0:0) | | ↑ |
| 4 | 380.147 | 7.33E-23 | 0.877  (0.854-0.900) | S-Lactoylglutathione | | ↓ |
| 5 | 425.418 | 0.001576 | 0.734  (0.697-0.781) | Octacosanoic acid | | ↓ |
| 6 | 504.483 | 0.000447 | 0.718  (0.687-0.758) | Cer(d18:1/12:0) | | ↓ |
| 7 | 527.117 | 0.000339 | 0.704  (0.676-0.754) | Raffinose | | ↓ |
| 8 | 572.388 | 0.000154 | 0.707  (0.673-0.750) | LysoPC(20:1(11Z)/0:0)/  LysoPC(22:4(7Z,10Z,13Z,16Z)/0:0)/  PC(18:1(9Z)e/2:0) | | ↓ |
| 9 | 609.378 | 0.000104 | 0.688  (0.650-0.726) | Torvoside G | | ↓ |
| 10 | 645.602 | 0.001111 | 0.745  (0.712-0.781) | CE(16:1(9Z)) | | ↑ |
| 11 | 693.347 | 0.001197 | 0.694  (0.664-0.731) | all-trans-Heptaprenyl diphosphate | | ↓ |
| 12 | 720.528 | 0.002648 | 0.675  (0.644-0.712) | PC(15:0/16:0) | | ↓ |
| 13 | 766.023 | 0.000192 | 0.721  (0.690-0.778) | NADP | | ↓ |
| 14 | 973.878 | 0.001542 | 0.696  (0.667-0.737) | TG(20:0/20:0/20:1(11Z)) | | ↓ |

**Table S5.** Pathways regulated in TB patients and healthy controls.

| **Pathway Hit** | **Raw p** | **-Log10 (p)** | **Impact** |
| --- | --- | --- | --- |
| Glycerophospholipid metabolism | 0.034843 | 1.4579 | 0.11182 |
| Glycerolipid metabolism | 0.12665 | 0.8974 | 0.01402 |
| Ether lipid metabolism | 0.15591 | 0.80713 | 0.08434 |
| Sphingolipid metabolism | 0.16308 | 0.7876 | 0.26978 |
| Pyruvate metabolism | 0.1702 | 0.76905 | 0.0591 |
| Galactose metabolism | 0.20495 | 0.68836 | 0.03188 |
| Glutathione metabolism | 0.21173 | 0.67421 | 0.0018 |

**Table S6.** 4 m/z signals selected as metabolite features for differentiating RR-TB patients from RS-TB patients.

| **ID** | **m/z** | **P value** | **AUC** | **Potential**  **biomarkers** | **Regulated expression** |
| --- | --- | --- | --- | --- | --- |
| 1 | 164.012 | 0.011495 | 0.821 (0.794-0.859) | Taurine | ↑ |
| 2 | 174.048 | 0.012624 | 0.802 (0.768-0.829) | Homocysteine | ↑ |
| 3 | 207.077 | 0.048588 | 0.829 (0.806-0.857) | Uric acid | ↑ |
| 4 | 214.952 | 0.009244 | 0.863 (0.836-0.894) | Ascorbic acid | ↑ |

**Table S7.** Comparison of the diagnostic performance of RR-TB using various data inputs and algorithms based on biomarkers.

| **Algorithm** | **Cohorts** | **AUC (95% CI)** | **Sensitivity** | **Specificity** |
| --- | --- | --- | --- | --- |
| LR | Validation | 0.857 (0.806-0.891) | 0.875 | 0.857 |
|  | Discovery | 0.658 (0.614-0.703) | 0.647 | 0.692 |
| PLS | Validation | 0.786 (0.746-0.819) | 0.750 | 0.857 |
|  | Discovery | 0.663 (0.612-0.712) | 0.647 | 0.654 |
| RF | Validation | 0.857 (0.817-0.889) | 0.750 | 1.000 |
|  | Discovery | 0.998 (0.996-0.999) | 1.000 | 0.962 |
| SVM | Validation | 0. 821 (0.781-0.857) | 0.875 | 0. 857 |
|  | Discovery | 0.661 (0.623-0.699) | 0.647 | 0.692 |

**Table S8.** Pathways regulated in RR-TB patients and RS-TB patients.

| **Pathway Hits** | **p** | **-Log10 (p)** | **Impact** |
| --- | --- | --- | --- |
| Taurine and hypotaurine metabolism | 0.015 | 1.8121 | 0.429 |
| Cysteine and methionine metabolism | 0.063 | 1.2037 | 0.138 |
| Primary bile acid biosynthesis | 0.086 | 1.0631 | 0.008 |
| Purine metabolism | 0.121 | 0.9184 | 0 |
